# CLIP knows image aesthetics

**DOI:** 10.3389/frai.2022.976235

**Published:** 2022-11-25

**Authors:** Simon Hentschel, Konstantin Kobs, Andreas Hotho

**Affiliations:** Chair of Data Science, Institute of Computer Science, Julius-Maximilians-Universität of Würzburg, Würzburg, Germany

**Keywords:** Image Aesthetic Assessment, CLIP, language-image pre-training, text supervision, prompt engineering, AVA

## Abstract

Most Image Aesthetic Assessment (IAA) methods use a pretrained ImageNet classification model as a base to fine-tune. We hypothesize that content classification is not an optimal pretraining task for IAA, since the task discourages the extraction of features that are useful for IAA, e.g., composition, lighting, or style. On the other hand, we argue that the Contrastive Language-Image Pretraining (CLIP) model is a better base for IAA models, since it has been trained using natural language supervision. Due to the rich nature of language, CLIP needs to learn a broad range of image features that correlate with sentences describing the image content, composition, environments, and even subjective feelings about the image. While it has been shown that CLIP extracts features useful for content classification tasks, its suitability for tasks that require the extraction of style-based features like IAA has not yet been shown. We test our hypothesis by conducting a three-step study, investigating the usefulness of features extracted by CLIP compared to features obtained from the last layer of a comparable ImageNet classification model. In each step, we get more computationally expensive. First, we engineer natural language prompts that let CLIP assess an image's aesthetic without adjusting any weights in the model. To overcome the challenge that CLIP's prompting only is applicable to classification tasks, we propose a simple but effective strategy to convert multiple prompts to a continuous scalar as required when predicting an image's mean aesthetic score. Second, we train a linear regression on the AVA dataset using image features obtained by CLIP's image encoder. The resulting model outperforms a linear regression trained on features from an ImageNet classification model. It also shows competitive performance with fully fine-tuned networks based on ImageNet, while only training a single layer. Finally, by fine-tuning CLIP's image encoder on the AVA dataset, we show that CLIP only needs a fraction of training epochs to converge, while also performing better than a fine-tuned ImageNet model. Overall, our experiments suggest that CLIP is better suited as a base model for IAA methods than ImageNet pretrained networks.

## 1. Introduction

Automatically assessing the aesthetics of an image (Image Aesthetics Assessment, IAA) is useful for tasks like choosing the most beautiful image from a set of photos (Lennan, 2018), sorting an image collection (Google, 2021), or image editing (Fischer et al., 2020). IAA methods based on deep neural networks are usually built on top of models that were trained on the ImageNet classification task (Ma et al., 2017; Sheng et al., 2018; Talebi and Milanfar, 2018; Hosu et al., 2019; Li et al., 2020; Zeng et al., 2020; Ke et al., 2021). Like previous work (Pfister et al., 2021), we argue that content classification is not well-suited as a pretraining task for IAA methods, since the model is optimized to be invariant to important factors of image aesthetics, e.g., contrast, lighting, or composition, as these are not important to identify the content of an image.

In contrast to content classes, natural language can provide much richer descriptions. Among others, it can describe styles (“a high-contrast image of a dog”, “a black-and-white portrait”), compositions (“a man standing next to a chair”, “an image of a house with the sun in the upper right corner”), or can even directly express the subjective feeling of aesthetics (“a beautiful sunset”, “an ugly sweater”). We hypothesize that models trained using natural language supervision are better suited for the IAA task, since they extract broader and more useful features. In this paper, we utilize the Contrastive Language-Image Pretraining (CLIP) (Radford et al., 2021) model for our experiments.

CLIP consists of an image encoder and a text encoder network, both transformer models (Vaswani et al., 2017; Dosovitskiy et al., 2021). It is trained on a large corpus of web images and their corresponding descriptions. Images and texts are mapped to a 512-dimensional vector space using the image and text encoder, respectively. Both encoders are trained to maximize cosine similarity between corresponding images and texts while minimizing similarity between mismatching image-text pairs. Due to this training objective, CLIP is able to classify images based on natural language prompts: Given an image and a set of possible text descriptions, the image and all texts are encoded using CLIP's image and text encoder, respectively. Then, the cosine similarities between each text embedding and the image are computed. The text prompt with the highest similarity is chosen as the predicted class. CLIP shows very good performance on many datasets in a kind of “zero-shot” setting; no training example from the target dataset is used and only suitable natural language prompts are engineered to identify the correct class. In the original paper, CLIP's capabilities only have been shown on *content classification* datasets (Radford et al., 2021). For the IAA task, however, the extraction of both, *content and style* features is important, so it is not obvious that CLIP performs well on this task. We hypothesize that, due to the language guided training objective, CLIP's image encoder also extracts useful features for the IAA task, such as lighting, composition, and properties of beauty ideals, such that it serves as a better base model for IAA networks than commonly used ImageNet classification models.

**Figure 1:**
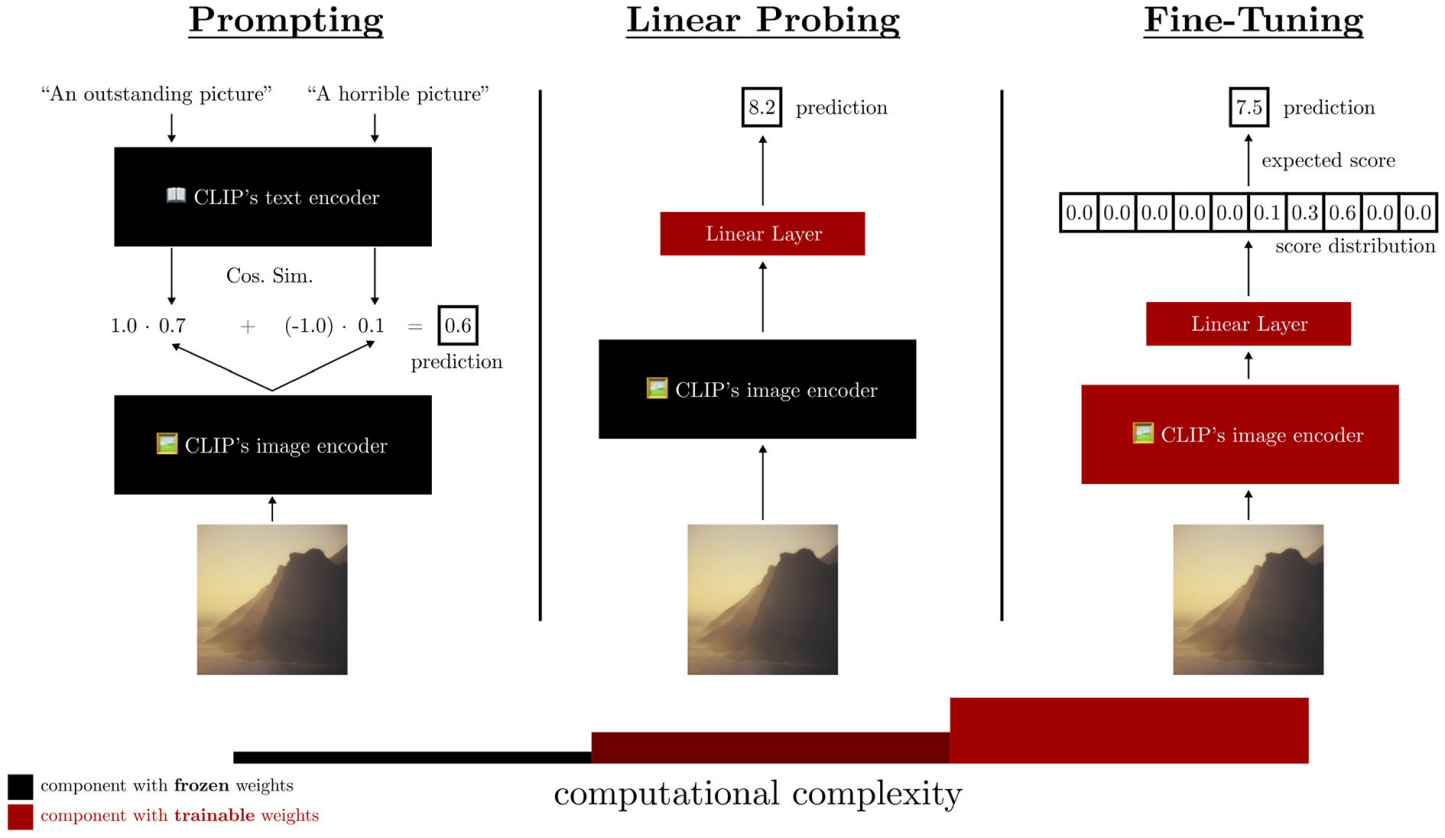
A schematic overview of our investigation on the suitability of CLIP as a base model for the Image Aesthetic Assessment task. The three steps increase in computational complexity. First, prompting does not modify any parameters. Second, linear probing trains a linear regression on top of the last hidden activations of CLIP's image encoder. Third, we fine-tune all parameters of the model on the AVA dataset. Our experiments show that CLIP is suitable as a base model for IAA methods, since it extracts features related to image aesthetics.

To test our hypothesis, we conduct a three-step study to investigate the suitability of CLIP for the IAA task. As a ground truth for this very subjective task, we utilize the AVA dataset and estimate CLIP's performance on the IAA binary classification task (“aesthetic”/“not aesthetic”) and ranking of images (predicting a mean aesthetic score over user ratings). In each of the three steps, we increase the computational complexity, i.e., the number of parameters trained for the method. A schematic overview of our investigation is shown in Figure 1.

First, inspired by CLIP's ability to classify images without explicit training using only natural language prompts (Radford et al., 2021), we test multiple ways of prompting CLIP to estimate the aesthetic appeal of images. We use fixed prompts, add context to better reflect the content of images, and create ensembles of prompts for binary predictions. To overcome the issue that only classification tasks can be solved by finding the most similar text prompt, we introduce a method to convert multiple positive and negative prompts to a continuous score by calculating a weighted sum over the prompt similarities. Our results show that plausible, carefully chosen prompts can beat simple baselines without any model training, which shows that CLIP extracts features correlated with subjective adjectives such as “outstanding” or “horrible”.

Second, we use CLIP's image encoder as a static feature extractor and train a linear regression on top of it, called Linear Probing (Radford et al., 2021). We compare the results to the same regression optimized on features extracted from a comparable Vision Transformer (ViT) (Dosovitskiy et al., 2021), which has been pretrained on the ImageNet classification task. We show that CLIP's features substantially outperform ImageNet features, indicating that they extract more useful information for the IAA task from the image. In fact, Linear Probing CLIP achieves a performance competitive to fully fine-tuned ImageNet models, while only optimizing 768 model parameters.

Finally, following most IAA methods (Ma et al., 2017; Sheng et al., 2018; Talebi and Milanfar, 2018; Hosu et al., 2019; Zeng et al., 2020), we fine-tune both the ImageNet ViT and CLIP's image encoder on the AVA training dataset. We can show that CLIP performs better than the ImageNet model while needing only a fraction of the training epochs to converge. Also, the fine-tuned CLIP is competitive to state-of-the-art IAA models that usually use more complicated training procedures and architectures (Ma et al., 2017; Hosu et al., 2019; Zeng et al., 2020; Ke et al., 2021). Overall, our investigations show that CLIP is well-suited as a base model for the IAA task.^1^

Our contributions in this work are threefold:

- We show that CLIP can solve the IAA task using only plausible text prompts.
- To this end, we propose a method that is able to predict continuous scores using only text prompts.
- Finally, we are the first to show that CLIP is a good feature extractor and base model for the IAA task, converging faster and performing better than a comparable ImageNet-pretrained Vision Transformer.

The rest of the paper is structured as follows: Section 2 introduces the task, the data used for our experiments, and the overall experimental setting. Section 3 discusses related works. In Section 4, we conduct our experiments using prompting (Section 4.1), linear probing (Section 4.2), and fine-tuning (Section 4.3). Sections 5 and 6 discuss and conclude the work, respectively.

## 2. Task and setting

We work on the task of Image Aesthetic Assessment (IAA), i.e., automatically quantifying the aesthetic appeal of an image. We evaluate all models with the common benchmark dataset AVA (Murray et al., 2012). It consists of 255,522 (229,971 training and 25,551 test) images scraped from a photography website^2^ along with the distribution of user ratings in {1, 2, …, 10}. The mean score is then used as the indicator for how aesthetically pleasing the image is (higher is better).

In the literature, mainly two tasks can be found (Talebi and Milanfar, 2018; Hosu et al., 2019; Li et al., 2020; Zeng et al., 2020; Ke et al., 2021). The *binary task* separates the dataset into aesthetic and unaesthetic images based on a threshold (typically being five). A model is then trained to assign each image to one of the two classes. Accuracy is used to evaluate model predictions.

The more realistic task is the *continuous task*, since it allows for applications such as image ranking or generally a comparison between different images (Fischer et al., 2020; Google, 2021). Here, the model has to output a continuous value, higher values indicating more aesthetic images. As evaluation metrics, Pearson and Spearman correlations are computed between the predictions and ground truth mean scores. While Pearson indicates the linear correlation between the values, Spearman measures the correctness of the image ranking. In our experiments, we report all three evaluation metrics to be able to compare to different related and previous works.

For our experiments, we always use CLIP's “ViT-B/32” variant for the image encoder, which means that images are resized and center-cropped to 224 × 224 pixels, split into non-overlapping patches of 32 × 32 pixels, and fed through 12 transformer layers with a hidden size of 768, MLP size of 3,072, and 12 attention heads in each multihead attention. Larger image encoders have been released by Radford et al. (2021), but we focus on the ViT-B/32 variant, since it is fast and fulfills the purpose of this paper's research question: “Does CLIP extract features that can be used for Image Aesthetic Assessment?”

## 3. Related work

Many different deep learning models have been proposed for predicting the aesthetic quality of images (Ma et al., 2017; Sheng et al., 2018; Talebi and Milanfar, 2018; Hosu et al., 2019; Li et al., 2020; Zeng et al., 2020; Ke et al., 2021). Several architectures and loss functions have been developed in recent years and can often be interpreted as ways to getting the model to learn better features to solve the task, thus leading to improved performance on the binary and continuous IAA tasks.

NIMA (Talebi and Milanfar, 2018) replaces the classification head of an ImageNet pretrained convolutional neural network (CNN) with a fully connected layer and is fine-tuned to predict the image's rating distribution. The Earth Mover's Distance (EMD) loss explicitly guides the network to include the order of scores in the training process. Even though simple and elegant, this approach achieves competitive performance on both tasks, compared to more advanced methods.

PA_IAA (Li et al., 2020) uses multi-task learning to not only predict a general but also a personalized aesthetics score based on individual personality traits. The authors train a Siamese network based on pretrained classification models using additional personality training data and the EMD loss. This way, the network learns features that model personality and subjectivity. Note that for this approach, additional personalized data is necessary, which is not present in the AVA dataset.

Most methods resize and crop images to fit them into the required dimensions of the underlying architecture, which can lose details and destroy image compositions. MLSP (Hosu et al., 2019) allows the model to extract features from the whole image by using activations from multiple convolution blocks of a pre-trained Inception network. On these features, the authors train a custom CNN architecture.

All mentioned models and most models from the literature rely on convolutional architectures which have been pre-trained on the ImageNet dataset (Ma et al., 2017; Sheng et al., 2018; Talebi and Milanfar, 2018; Hosu et al., 2019; Li et al., 2020; Zeng et al., 2020; Ke et al., 2021). We argue that models trained on a classification task miss important features for the IAA task. For example, since the ImageNet classification task aims to predict the content class of the image, style features such as contrast or lighting conditions are not important. In fact, a model that can correctly identify the content of an image should not be influenced by environmental situations such as lighting or image filters. Based on this argument, Pfister et al. (2021) propose multiple pretraining tasks that are specifically designed to let the network extract features important for IAA. For this, they collect a large dataset of highly aesthetic images from a stock photo website and destroy these images using image filters. They then pretrain a MobileNet to distinguish between modified and clean images and let the model rank the degree of modification on the image. Fine-tuning the pretrained model on AVA then achieves better results than the fine-tuned classification model and converges faster.

Our work also tries to estimate the usefulness of better features for the IAA task. We explicitly investigate the use of CLIP as a static feature extractor and base model for fine-tuning IAA models.

## 4. Experiments

In this section, we present the experiments we conduct to investigate the usefulness of CLIP's features for the IAA task. We go from computationally simple to complex methods, i.e., prompting, linear probing, and fine-tuning CLIP. First, we show that by using natural language prompts, CLIP is able to estimate an image's aesthetic score better than simple baselines, indicating that CLIP has learned to correlate subjective text features with image features. The prompting method relies on a useful text encoder. In a second step, we optimize a linear regression on CLIP's image features and compare this to a linear regression model trained on image features from a classification Vision Transformer (ViT). We show that CLIP's features lead to substantially better performance and achieve competitive results to fully fine-tuned IAA models. Third, we compare the performance of a fine-tuned CLIP image encoder to a fine-tuned ImageNet classification ViT. Again, CLIP outperforms the ViT-based model while also converging in a fraction of the number of epochs. For each experiment, we demonstrate how each method can be used to predict binary but also continuous scores, allowing us to compare all methods on all evaluation metrics.

All experimental results are shown in Table 1. We compare our results with results reported in the literature. We train and test our methods on the same datasplit as MP_*ada*_ (Sheng et al., 2018), A-LAMP (Ma et al., 2017; Zeng et al., 2020), and MUSIQ (Ke et al., 2021) and compare our results to these methods. Note that PA_IAA (Li et al., 2020), NIMA (Talebi and Milanfar, 2018), and MLSP (Hosu et al., 2019) certainly train and validate their models on a different dataset split as the other models, thus their stated test results are only mentioned for reference. To allow a fair comparison, we display the results of a NIMA reimplementation (Lennan et al., 2018) that has been trained on the same datasplit as the other models. In addition, we train and evaluate MLSP on our datasplit using the code for the original implementation of MLSP provided by Hosu et al. (2019).

**Table 1:** Comparison between the results of all our models and the results of existing models, which were published in their respective papers.

**Method**	**Accuracy**	**Spearman**	**Pearson**
PA_IAA (Inception-V3) (Li et al., 2020)	0.837	0.677	n/a
NIMA (Inception-V2; paper results) (Talebi and Milanfar, 2018)	0.815	0.612	0.636
MLSP (InceptionResNet-V2; paper results) (Hosu et al., 2019)	0.817	0.756	0.757
Majority voting baseline	0.703	—	—
MP_*ada*_ (Sheng et al., 2018)	**0.830**	—	—
A-LAMP (Ma et al., 2017)	0.825	—	—
ResNet101 (Zeng et al., 2020)	0.808	0.719	0.720
MUSIQ (Ke et al., 2021)	0.815	0.726	0.738
NIMA (MobileNet; reimplementation) (Lennan et al., 2018)	n/a	0.626	0.609
MLSP (InceptionResNet-V2; original code trained and evaluated on our datasplit) (Hosu et al., 2019)	0.808	0.714	0.728
Fixed prompt CLIP	0.725	0.435	0.453
Context-aware prompt CLIP	0.737	0.539	0.554
Ensembling prompt CLIP	0.756	0.539	0.554
Linear probing ViT (ImageNet21k)	0.767	0.574	0.587
Linear probing CLIP	0.800	0.683	0.694
Fine-tuned ViT (ImageNet21k)	0.793	0.660	0.675
Fine-tuned CLIP	0.816	**0.731**	**0.741**

“n/a” refers to metrics not stated in the original work. Methods with “—” for Spearman and Pearson correlation are only designed to solve the binary task. Methods in gray rows do not state the used training/test split or do not use the same split as ours, thus we only reference them for context. In the case of NIMA, we refer to the results of a reimplementation trained on the correct datasplit (Lennan et al., 2018). For MLSP, we use the original code provided by Hosu et al. (2019) and train it on our datasplit. The other models use the same training/test split as our work.

Bold values indicate the best performance on the tested datasplit.

Finally, we also introduce a simple majority voting baseline that always predicts the positive class in the binary classification task. Since the distribution of labels is skewed toward aesthetic images, always predicting the positive class already gives 70.3 % accuracy. Due to no variation in the predictions, correlations cannot be computed, thus this baseline is not available for the continuous task.

### 4.1. Prompting

The experiments in this section aim to answer the question: “Does CLIP extract image features that are related to the way we express aesthetics using language?”

CLIP has been shown to have good “zero-shot” performance on classification tasks by prompting the network with text inputs based on the desired labels and measuring the similarity to the image's feature vector. More formally, CLIP's image encoder represents an input image (resized and center-cropped to 224 × 224 pixels) as a 512-dimensional vector *I*. CLIP's text encoder encodes all possible class labels ℂ, getting 512-dimensional vectors *T*_*i*_ for *i*∈ℂ. Often, string templates are used to embed the class labels into a coherent natural language text prompt before feeding it into CLIP's text encoder (Radford et al., 2021). The label whose vector has the highest cosine similarity to the image vector *argmax*_*i*∈ℂ_cos(*I, T*_*i*_) is chosen as the prediction.

It is important to note that there is no training involved in this method. Since CLIP was trained on natural language, any text label can be used for the classification task. In our setting, we use adjectives used to describe aesthetic or unaesthetic images as labels, e.g., “beautiful” or “ugly”. To collect possible labels, we search synonyms for “ugly” and “great” in WordNet (Princeton University, 2010) and augment the list of positive adjectives with “beautiful” and “pretty”, since these were not part of the list. Overall, we get 27 positive and 12 negative words.

Given two words for aesthetic or unaesthetic images, we can construct prompts by embedding them into a string template that are then used to measure the similarity to the image vector. We try different prompting methods, i.e., fixed prompts, prompts with context, and ensembling. All of the prompting methods are designed for classification tasks, thus are naturally applicable to the binary classification task of IAA. Predictions are made by finding the most similar prompt to the image. For the continuous task, however, it is necessary to predict a single scalar score that encodes both, positive and negative image aspects. We thus propose a simple but effective strategy to compute scores from prompts.

Intuitively, prompts that suit an image better should be more similar to the image's embedding, while non-fitting prompts are less similar. Thus, high similarity between a positive prompt and the image should lead to a high image aesthetic score. Correspondingly, if the negative prompt has high similarity, the score should get lower. We build on this intuitive idea and calculate the predicted score for an image and all prompts we use by weighting the similarity of each prompt to the image with one or minus one, depending on the prompt's incentive:


(1) $$\begin{array}{l} score\left(I\right)=\underset{i\in \mathbb{C}}{\sum }cos\left(I,T_{i}\right)\cdot w_{i} \end{array}$$

(2) $$w_{i}=\{\begin{array}{ll} 1, & \text{if }i\text{ is a positive label}\\ -1, & \text{if }i\text{ is a negative label} \end{array}.$$

Since the cosine similarity is in range [−1,1], very dissimilar negative (positive) prompts make the score prediction higher (lower). In the following, we describe the prompting methods we try.

#### 4.1.1. Fixed prompt

Our fixed prompt approach utilizes exactly two prompts, one for aesthetic images, one for unaesthetic images. Both prompts are formed using the string template “a [label] picture”, where [label] is either a positive or a negative word from our list of adjectives.^3^ Given these two prompts, we find the one more similar to the image using CLIP and predict its label for the binary classification task. The weighted score prediction described above is used for the continuous task.

We evaluate all combinations of positive and negative prompts from our collection of adjectives. The labels performing best on AVA's training dataset for the binary task are “outstanding” and “atrocious” for positive and negative prompts, respectively. For the continuous task, “outstanding” and “horrible” perform best on the training dataset based on the Spearman correlation.

Row “Fixed Prompt CLIP” in Table 1 shows that using these prompts already shows better performance than always predicting the majority class. On the continuous scale, the predictions have moderate correlations with the ground truth. This indicates that CLIP extracts features from images that correlate with descriptive adjectives such as “outstanding” or “horrible”.

#### 4.1.2. Context-aware prompts

Fixed prompts do not account for the content of the image. Instead of the generic prompt “a beautiful picture”, we hypothesize that it is better to include the content of the image, e.g., “a beautiful picture of a dog”. This specification of the text prompt moves the encoded prompt vectors closer toward the image vector, thus reducing noise in similarity measurements and maybe helping with the improvement on the IAA task. Since it is not known what is in the photo, we use the text descriptions of all 1,000 ImageNet (Russakovsky et al., 2015) classes.^4^ We then construct a prompt using the string template “a [label] picture, of a #[content class]_*i*_”, where [content class]_*i*_ is the *i*th class name of ImageNet. Including the content as a hashtag showed performance improvements in preliminary experiments and accounts for the internet-based origins of the dataset used to train CLIP.

Instead of two prompts as with the fixed prompt approach, we now have 2,000, i.e., 1,000 for each positive/negative label. The label of the closest text prompt to the image vector is used as the prediction in the binary task, while the weighed score is calculated across all prompts in the continuous setting.

On the training set, we find that “smashing”/“horrible” are the best labels to use for the binary task. In the continuous setting, the labels “outstanding”/“horrible” perform best. The results in Table 1 show that this method improves all evaluation metrics in contrast to the fixed prompt setting, especially in the continuous task.

**Figure 2:**
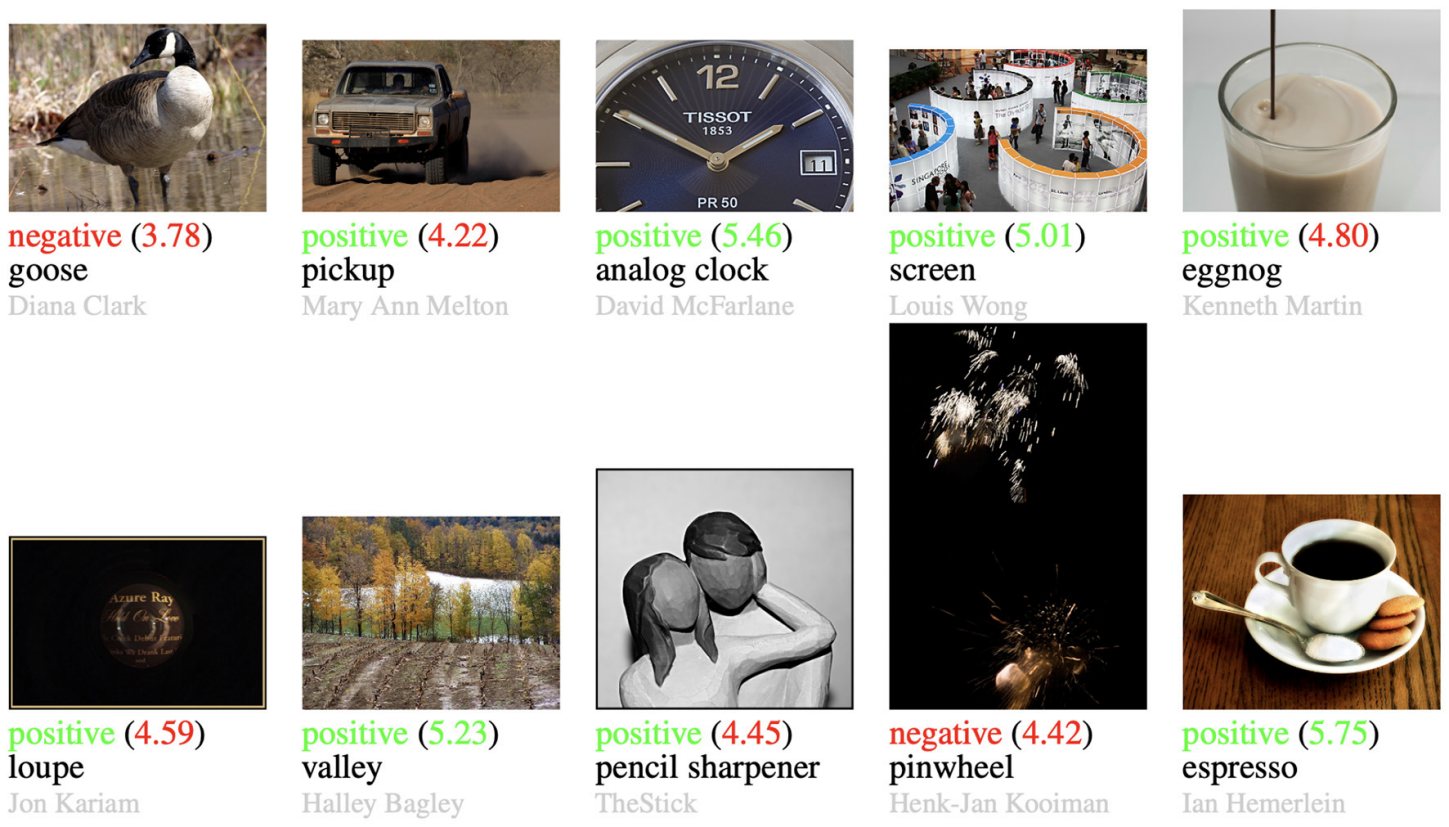
Example images with the binary prediction by the context-aware prompts for CLIP, the actual score by AVA (in parenthesis), and the content of the prompt with the highest similarity to the image. The predictions and actual scores are colored to easily find the predictions that are correct or wrong. Image credit is given in gray (name or username of photographer on dpchallenge.com).

To better understand the effects of adding content classes to the prompts, we visualize some test images with their binary prediction, their actual AVA mean score, as well as the content class that was used in the most similar prompt in Figure 2. We can observe that the closest content class tends to describe the content quite well or approximates its visual appearance. The predicted binary aesthetic label is mostly correct. Overall, the use of content classes improves the results by moving the text prompt vector closer to the image vector. Then, the choice between an aesthetic and an unaesthetic image is made depending on the content of the image.

#### 4.1.3. Ensembling

Our ensembling approach is structurally similar to the context-aware prompts. However, we condense the 2,000 prompts down to two vectors by averaging all prompt vectors of each aesthetic label. The best labels are the same as for the context-aware prompts, but the results in Table 1 show that this method improves the performance while being computationally less expensive, since only two instead of 2,000 comparisons have to be done for each image.

Our prompting results show that CLIP extracts features that can be used for the IAA task. These features correlate with text prompts describing the corresponding aesthetic value of the image. Overall, the labels “outstanding” and “horrible” are well-suited for this task. In an application, it might be possible to get acceptable results if only a pretrained CLIP model is available.

### 4.2. Linear probing

Most IAA models use a classification neural network pretrained on ImageNet (Deng et al., 2009). We now want to investigate the question whether CLIP's features (as taken from the pretrained model) are more suitable for the IAA task than features from an ImageNet classification model. Intuitively, CLIP extracts broader features than an ImageNet model, since for classification, only the content of the image is important. Features describing certain aspects of the image are not needed for classification, especially features like lighting, contrast, and other properties of the image that are important for IAA. On the other hand, CLIP's language-based training learns broader features, since language is more descriptive and thus more nuanced than class labels.

For the experiments in this section, we train a linear regression on the features of CLIP's image encoder to predict AVA's training images' mean score. We compare the results on AVA's testset with results obtained by using features from a Vision Transformer (ViT)^5^ (Wightman, 2019; Dosovitskiy et al., 2021) that is very similar in size to CLIP's image encoder (also of type “ViT-B/32”), but was trained on ImageNet21k (Ridnik et al., 2021). According to Radford et al. (2021), the architectural difference between ViT and CLIP's image encoder are additional layer normalizations and a slightly different initialization scheme. We thus argue that it is fair to compare both models.

For the linear regressions, we take the activations from the last layer before the output for both models. This way, both models provide 768-dimensional feature vectors. The output of the linear regression model is a scalar value, which is thresholded at five to predict binary classes while staying untouched for the continuous task.

As can be seen in Table 1, using CLIP as a static feature extractor is much more effective for the IAA task than the ImageNet model. It achieves approx. 3 % points higher accuracy and improves both correlations by approx. 0.1. Linear probing also significantly improves on the prompting approach. In fact, our results approach competitive performance to IAA models that optimize all network weights, such as the method introduced by Zeng et al. (2020). Their method fine-tunes a ResNet101 pretrained on ImageNet using the cross entropy loss, while also changing the target score distributions based on manual examination of AVA's target labels. Our results are obtained by simply minimizing the mean squared error of the predicted and target mean score. Using more sophisticated loss functions and target labels might improve the results while still only requiring few parameters to optimize.

**Figure 3:**
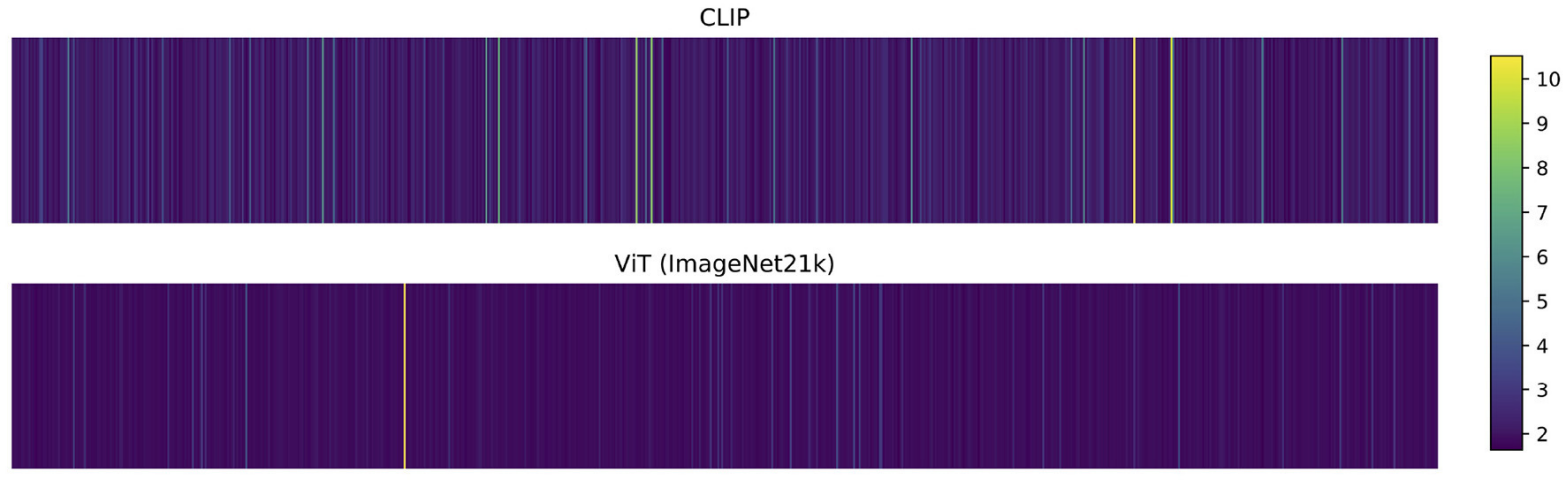
Visualized weights of the linear regression trained on AVA. While the ImageNet-pretrained Vision Transformer (ViT) uses mainly one feature in the estimation of the target score, CLIP's features are more broadly used. Together with the fact that the linear regression on CLIP features outperforms ImageNet features, this indicates that CLIP's image features encode more useful and broader information for the IAA task.

To better understand the difference between CLIP's and ViT's features, we visualize the 768 weights from the trained linear regression in Figure 3. For this, we scale the linear regression weights by the standard deviation of the input features to alleviate the impact of differences in the features' value ranges. The visualization shows that the linear regression mainly focuses on only one dimension of the ViT features but on a broader set of features from CLIP. Thus, the features from CLIP are a better starting point to predict the aesthetic score of images, since the linear regression model can combine more useful features. Overall, we can summarize that CLIP as a static feature extractor is better suited for the IAA task than a classification based feature extractor.

### 4.3. Fine-tuning

Our final experiment poses the question, whether CLIP is a suitable base for fine-tuning on the IAA task. Since we know from the previous experiment that CLIP's embedding vectors are better suited for IAA than ImageNet features, we hypothesize that CLIP's image encoder is a better starting point for fine-tuning and should train faster and perform better on the IAA task.

We thus fine-tune CLIP and the Vision Transformer on the AVA training dataset. We replace the last layer of both models with a linear layer with 10 outputs, each representing one possible score. A softmax activation function converts the outputs into distributions that can be compared to the target score distribution. We employ the EMD loss as proposed by NIMA (Talebi and Milanfar, 2018) and fine-tune the models using a batch size of 128, the SGD optimizer with momentum of 0.5 and a learning rate of 0.01 for the classifier head and 0.0001 for the rest of the model. Besides resizing and center-cropping the images to 224 × 224 pixels, no data augmentation is applied. Early stopping trains the model until the loss on a 10 % subset of the training data does not improve for 10 consecutive epochs.

The results in Table 1 show that the fine-tuned CLIP model performs substantially better than the ImageNet model. Most notably, the CLIP model achieves the highest Pearson and Spearman correlations for the models trained and evaluated on the same dataset split as ours. It also achieves the best Accuracy when only compared to methods that are designed to output continuous scores. Compared to the previous experiment, both models improve on their linear probing counterpart. However, Linear Probing CLIP achieves better performance on all metrics than the fine-tuned ImageNet model while optimizing a fraction of the parameters.

In addition to the improved performance, we find that the fine-tuned CLIP model converges much faster than the ImageNet model. The best training epoch for the CLIP fine-tuning is 35, while ViT needs more than seven times longer and achieves the best loss after 247 epochs. This behavior has also been observed by Pfister et al. (2021), who found that models pretrained on tasks that promote the learning of useful features for the IAA task convergence more quickly than ImageNet pretrained models.

In conclusion, it is fair to state that the CLIP model is a useful base for fine-tuning to the IAA task, since it extracts a broader set of features from images than comparable classification based models. In addition to better performance, faster training convergence is a property that can accelerate IAA research.

## 5. Discussion

Our systematic experiments have shown that CLIP performs consistently better than a comparable Vision Transformer trained on ImageNet21k. A linear regression trained on CLIP's representations even outperforms a fully fine-tuned ImageNet-ViT. We hypothesize that the language-enriched training data is responsible for the better performance. An alternative hypothesis would be that the improvements stem from the amount of data CLIP has been trained on (400 million image-text pairs) compared to the ImageNet-ViT (approximately 14 million images). To rule this out, we compare a ResNet-50 (He et al., 2016) trained on ImageNet21k to ResNet-50 image encoders from OpenCLIP (Ilharco et al., 2021) trained on the Conceptual 12M (Changpinyo et al., 2021) and the YFCC-15M (a subset of the YFCC-100M dataset; Thomee et al., 2016) datasets. These datasets comprise of approx. 12 and 15 million image-text pairs, respectively, so are comparable in size to the ImageNet21k dataset. The results for the linear probing of these models in Table 2 show that both CLIP models show better performance than the ImageNet21k model. This provides more evidence that the dataset size is not the reason for CLIP's performance improvement, but the quality of the training data and its training task.

**Table 2:** Linear probing results for similar ResNet-50 (He et al., 2016) models.

**Method**	**Num. training images**	**Accuracy**	**Spearman**	**Pearson**
ResNet-50 (ImageNet21k)	Approx. 14 million	0.758	0.554	0.567
ResNet-50 OpenCLIP (Conceptual 12M)	Approx. 12 million	0.777	0.623	0.631
ResNet-50 OpenCLIP (YFCC-15M)	Approx. 15 million	0.793	0.662	0.673
ResNet-50 CLIP (OpenAI)	Approx. 400 million	0.793	0.674	0.682

CLIP models trained on similarly sized datasets as ImageNet21k still outperform the baseline model. The performance of CLIP on AVA improves with larger dataset sizes.

Besides the comparison to ImageNet models, our experiments have also shown that CLIP can even perform better than the majority baseline when only using text prompts, which shows that CLIP has learned image and text features that are linked to image aesthetics. While Radford et al. (2021) have shown that CLIP is applicable to content classification tasks, the usefulness of CLIP for tasks that require the extraction of stylistic image features such as lighting, contrast, or composition, such as IAA, were not shown in the original paper. We have provided experimental evidence that CLIP is suited as a static and fine-tuned feature extractor for the IAA task.

**Figure 4:**
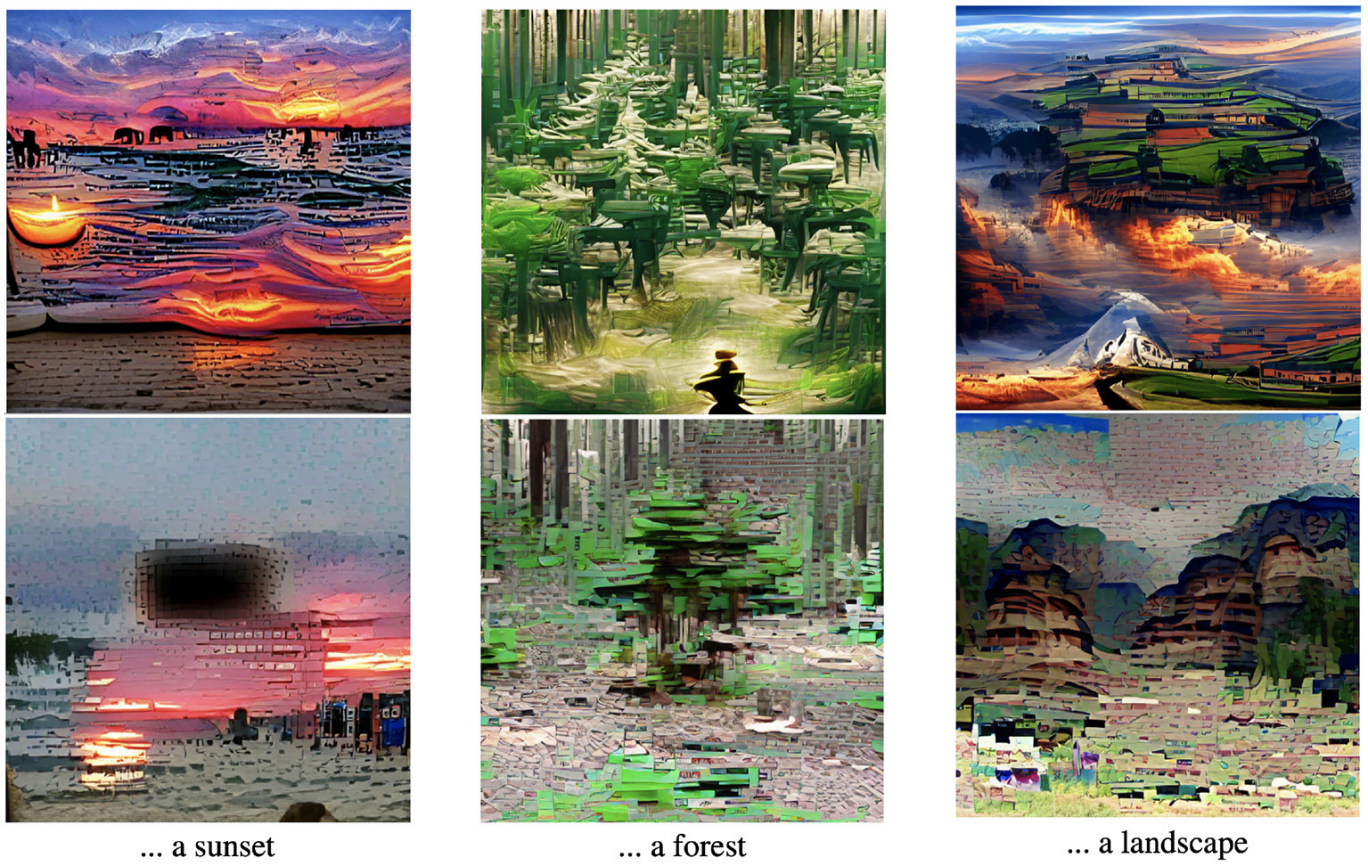
Example images generated by CLIP (Radford et al., 2021) and VQ-GAN (Crowson, 2022; Esser et al., 2021). The **top** images use a prompt of the form “A outstanding picture of...” while the **bottom** images use prompts of the form “A horrible picture of...”

While useful for IAA, the features related to image aesthetics extracted by CLIP also have an impact on tasks like CLIP-guided image generation (Snell, 2021). Image generation using CLIP optimizes an image (or the input to an image generator) such that CLIP's image encoder outputs a similar vector as an encoded text prompt. Recently, the AI art community found interesting prompting tricks by adding additional texts to the end of the text prompt (Dwyer, 2021; Snell, 2021). Here, correlations between text patterns and aesthetically pleasing (“trending on artstation”, “top of /r/art”) or very realistic images (“rendered with unreal engine”) in CLIP's training data are exploited. Our experimental results using prompts to let CLIP classify images as either aesthetic or unaesthetic show that it might also be possible to simply add positive adjectives in the text prompt to generate more aesthetic images. Figure 4 shows three example prompts where we generate images using a VQ-GAN (Esser et al., 2021), guided by a CLIP prompt^6^. Using positive adjectives (“A outstanding picture of...”) produce more vividly colored images with more contrast than using negative prompts (“A horrible picture”). This provides more evidence that CLIP knows image aesthetics and links the correct adjectives to corresponding image features.

In our prompting experiments, context-aware ensemble prompts work best. Since we do not know the correct content of the image, we have used the class names of the thousand ImageNet classes. While this seems to work quite well, it is not clear if the chosen class names are the best choice for this task. Since each ensemble is represented by only one feature vector, more content descriptions could be added without larger computational requirements during inference. Future work might evaluate more and different content descriptions, e.g., all class names of the ImageNet21k dataset or using Knowledge Graphs such as ConceptNet (Speer et al., 2018). Automatically generating image captions for the image content and using these for more targeted prompting is also an interesting, though more computationally expensive, research direction (Mokady et al., 2021).

Our CLIP training, especially the fine-tuning, is relatively simple compared to more advanced IAA methods (Ma et al., 2017; Hosu et al., 2019; Zeng et al., 2020; Ke et al., 2021). We hypothesize that using the methods from other state-of-the-art papers, but replacing ImageNet-pretrained models with CLIP's image encoder, will result in better performance and faster convergence as it does in our experiments. This paper restricts itself to provide an analysis of the usefulness of CLIP for the IAA task, so testing our hypothesis can be considered future work.

Our experiments exploring different computationally complex methods show that CLIP can be used with nearly zero as well as high computational overhead. The performance improves with the number of trained parameters, but using prompt ensembles alone already shows acceptable performance for many real-world tasks. Given that CLIP shows good performance on many different datasets without explicit training (Radford et al., 2021), we see high potential for CLIP in image databases, such as personal photo collections. It may be possible to store CLIP features for each image and use them to perform tasks like searching for contents using text (Baldrati et al., 2021), finding similar images, or creating image descriptions (Mokady et al., 2021). Our work shows that finding aesthetically pleasing pictures is another task that can be done with a pretrained CLIP model.

## 6. Conclusion

In this paper, we have investigated the suitability of CLIP, a model jointly trained with image-text pairs, as a (static) feature extractor or base model for the Image Aesthetic Assessment task. We have tested multiple methods with different levels of computational complexity, namely prompting, linear probing, and fine-tuning. All experiments have led to the conclusion that CLIP extracts features from images that are related to human's image aesthetic perception due to its training on images and their corresponding human-generated descriptions. In our experiments, CLIP features always outperform features extracted from an ImageNet classification model, which is the base model in most IAA papers. Given our results, we promote the use of CLIP as a base model for IAA tasks.

## Data availability statement

Publicly available datasets were analyzed in this study. This data can be found at: https://github.com/imfing/ava_downloader.

## Author contributions

KK designed the experiments and wrote the paper. SH performed the experiments and wrote the paper. AH critically revised the paper and experiments to meet high research standards. All authors contributed to the article and approved the submitted version.

## Conflict of interest

The authors declare that the research was conducted in the absence of any commercial or financial relationships that could be construed as a potential conflict of interest.

## Publisher's note

All claims expressed in this article are solely those of the authors and do not necessarily represent those of their affiliated organizations, or those of the publisher, the editors and the reviewers. Any product that may be evaluated in this article, or claim that may be made by its manufacturer, is not guaranteed or endorsed by the publisher.

## Footnotes

^1^Code will be made available at: https://github.com/shiemn/CLIP-knows-IA.

^2^ www.dpchallenge.com

^3^In preliminary experiments, we found that only giving the adjectives as prompts did not work well and “picture” in the prompt string template usually performed better than other words like “photo” or “image”.

^4^Class names from https://gist.github.com/yrevar/942d3a0ac09ec9e5eb3a, using only the name up to the first comma for each class.

^5^The pre-trained model can be downloaded with the timm python library under the model name “vit_base_patch32_224_in21k”.

^6^ https://colab.research.google.com/github/asdsadadad/BasicSR/blob/master/
